# Development and Initial Validation of the Exercise Behavioral Inconsistency Scale (ExBIS): Factor Structure, Reliability, Measurement Invariance, and Known-Groups Validity

**DOI:** 10.3390/bs16081308

**Published:** 2026-08-01

**Authors:** Bekir Erhan Orhan

**Affiliations:** Faculty of Sports Sciences, Istanbul Aydın University, Istanbul 34295, Türkiye; bekirerhanorhan@aydin.edu.tr

**Keywords:** exercise, intention–behavior gap, cognitive dissonance, rationalization, scale development, psychometric validation, measurement invariance

## Abstract

Many adults hold exercise intentions they do not enact, yet no instrument captures the cognitive–affective experience of this gap. This study developed and validated the Exercise Behavioral Inconsistency Scale (ExBIS), which measures four facets as a profile rather than a summative trait—intention level (IL), intention-action gap (IAG), emotional dissonance (ED), and internal rationalization (IR). Adults in Türkiye (*N* = 594; 57.9% male; *M*_age_ = 21.02 years) completed a 20-item Turkish pool, split into exploratory and confirmatory analyses (*n* = 297 each); 5 items were removed, yielding 15 items. Parallel analysis and the minimum average partial test supported four factors explaining 67.1% of variance (loadings 0.56–0.96). Confirmatory analysis replicated the structure, χ^2^(84) = 187.90, CFI = 0.954, RMSEA = 0.065, SRMR = 0.057, outperforming simpler alternatives. Neither a second-order nor a bifactor model supported a general factor (ECV = 0.37, ω_H_ = 0.30), so scores form a four-facet profile rather than a total, clustering weakly into IL/ED and IAG/IR. The McDonald’s omega was 0.78–0.90, and full scalar invariance held across gender and exercise-frequency groups. Subscales discriminated frequency groups as hypothesized (ω^2^ = 0.031–0.081). These cross-sectional data come from a young Turkish convenience sample surveyed in one language, so replication is required.

## 1. Introduction

Insufficient physical activity remains one of the most persistent public health problems, with more than a quarter of the world’s adult population failing to meet recommended activity levels (Guthold et al., 2018; Strain et al., 2024). This shortfall persists despite broad awareness of the benefits codified in international guidelines (Bull et al., 2020; World Health Organization, 2022). A striking feature of the problem is that it is not primarily a deficit of motivation. Most insufficiently active adults report intending to exercise, yet intention accounts for only a minority of the variance in subsequent behavior (Feil et al., 2023; Webb & Sheeran, 2006). Specifically in the physical activity domain, approximately half of intenders fail to enact their exercise intentions (Feil et al., 2023; Rhodes & de Bruijn, 2013). This discrepancy is known as the intention–behavior gap (Englert et al., 2023; Sheeran, 2002; Sheeran & Webb, 2016). It has shifted theoretical attention from models that culminate in intention formation, such as the theory of planned behavior, toward post-intentional accounts of action control that ask what happens after a person has decided to act (Ajzen, 1991; Rhodes, 2017, 2021; Rhodes & Yao, 2015).

Existing measurement practice has not kept pace with this theoretical shift. The gap itself is typically operationalized behaviorally, by cross-classifying stated intention against subsequent activity, an approach that identifies who is inconsistent but says nothing about the psychological experience of being inconsistent (Feil et al., 2023; Rhodes & de Bruijn, 2013). Cognitive dissonance theory predicts that holding a valued intention while repeatedly failing to enact it generates aversive arousal, and that individuals reduce this discomfort either by changing behavior or, more economically, by cognitive means such as trivializing the discrepancy, generating justifications, or recruiting external excuses (Cancino-Montecinos et al., 2020; Festinger, 1957; Harmon-Jones & Harmon-Jones, 2022; Harmon-Jones & Mills, 2019). Applied to exercise, this analysis suggests that the cognitive–affective architecture surrounding behavioral inconsistency is not a single quantity but a configuration of at least four distinguishable facets: the strength of the exercise intention itself (intention level), the perceived discrepancy between intention and enacted behavior (intention-action gap), the affective discomfort this discrepancy produces (emotional dissonance), and the justificatory cognitions deployed to neutralize it (internal rationalization). These facets are not assumed to converge on a single summable dimension; on the contrary, two of them index the commitment that makes a lapse aversive (intention level, emotional dissonance) and two index the gap and its neutralization (intention–action gap, internal rationalization), so the construct is better represented as a profile than as a total. Because such inconsistency is among the most frequently reported obstacles to sustained exercise participation, an instrument that captures its psychological structure has direct relevance for exercise and sport settings (Feil et al., 2023; Gabay et al., 2023).

Measures derived from the theory of planned behavior assess the strength of intention, but not its violation; action-control taxonomies classify behavioral profiles but not their affective and justificatory sequelae; and dissonance measures developed in other domains do not reference the recurring, self-relevant character of exercise lapses. The absence of such an instrument constrains research in two ways. Substantively, it prevents tests of whether dissonance and rationalization function as the maintenance mechanisms of chronic inconsistency that post-intentional theory implies, a hypothesis that a validated multidimensional instrument is a precondition for testing, but that the present cross-sectional design cannot itself adjudicate (Rhodes, 2017, 2021). Practically, it deprives interventionists of a candidate profile for identifying “unsuccessful intenders”, whose characteristics of high intention, high gap and high rationalization may mark them as candidates for action-planning and self-regulatory support rather than motivational enhancement (Englert et al., 2023; Sniehotta et al., 2005), a possibility that requires prospective predictive evidence before it can guide practice.

Three questions motivate the present instrument, none of which existing measures can answer. First, what is the internal cognitive–affective structure of exercise inconsistency, intending yet failing to exercise—a single quantity, or a configuration of separable facets? Behavioral cross-classification of intenders and actors identifies who is inconsistent, but is silent on this structure. Second, do the affective and justificatory responses to a lapse (emotional dissonance, internal rationalization) function as distinct, measurable components alongside intention strength and the enacted gap, as post-intentional and dissonance accounts imply, but which existing scales built to gauge intention strength, to classify behavioral profiles, or to index dissonance in unrelated domains were never designed to capture jointly? Third, can these facets be assessed as a profile that differentiates individuals who share the same behavioral gap but differ in how they experience and neutralize it—precisely the distinction that would motivate matching “unsuccessful intenders” to self-regulatory rather than motivational support? The unique contribution of the Exercise Behavioral Inconsistency Scale (ExBIS) is to render this four-facet configuration measurable within a single instrument, thereby converting a theoretically central but previously unmeasured construct into a testable one. Establishing that the configuration is psychometrically defensible is the necessary first step and the aim of the present study.

**H1:**  *ExBIS items would conform to a correlated four-factor structure (intention level (IL), intention–action gap (IAG), emotional dissonance (ED), and internal rationalization (IR), replicable across independent subsamples and not reducible to a single general factor.*

**H2:**  *Each subscale would show adequate internal consistency (ω and α ≥ 0.70).*

**H3:**  *The four-factor model would display at least scalar invariance across gender and across exercise frequency groups.*

**H4:**  *Subscale scores would differentiate exercise frequency groups, with IL and ED increasing and IAG and IR decreasing as exercise frequency increases.*

**H5:**  *IL and ED would correlate positively, and IAG and IR negatively, with exercise frequency, session duration, and exercise experience.*

## 2. Materials and Methods

### 2.1. Participants and Procedure

The participants were 594 adults recruited from across Türkiye by convenience sampling. Eligibility required being at least 18 years of age and reporting current engagement in, or a current intention to engage in, regular exercise; 36 respondents younger than 18 were excluded before analysis. The final sample (344 men, 57.9%; 250 women, 42.1%) had a mean age of 21.02 years (*SD* = 4.87, range 18–58). Most participants held or were pursuing a bachelor’s degree (57.6%); 30.0% reported high school education, 9.1% an associate degree, and 3.4% postgraduate qualifications. Reported weekly exercise frequency was 1–2 days for 29.5%, 3–4 days for 47.6%, and ≥5 days for 22.9% of the sample (Table 1). Data were collected online via Google Forms (Google LLC, Mountain View, CA, USA), with the survey link distributed through social media, following written informed consent. The questionnaire used a forced-response format, which precluded item-level missingness but cannot, by itself, guard against inattentive completion; accordingly, careless responding was screened post hoc (see Section 2.4 and Section 3.1). The study was approved by the Social and Human Sciences Ethics Committee of Istanbul Aydın University (decision no. 2026/01, 21 January 2026) and conducted in accordance with the Declaration of Helsinki. Because recruitment used convenience sampling via social media, the sample skews young and university-educated, and is not representative of the wider adult population (see Section Limitations and Future Directions for implications).

For structural analyses, the sample was randomly split (seed = 31,216) into a development subsample (Sample 1, *n* = 297) used for exploratory factor analysis and a validation subsample (Sample 2, *n* = 297) used for confirmatory factor analysis. The subsamples did not differ in age, *t*(592) = −1.05, *p* = 0.293, or gender composition, χ^2^(1) = 2.00, *p* = 0.158. Both subsamples exceed conventional adequacy benchmarks. Sample 1 approaches the “good” band for factor analysis, with a subject-to-item ratio near 20:1 that comfortably exceeds the recommended 10:1 (Comrey & Lee, 1992; Tabachnick & Fidell, 2019). It also remains adequate under communality-based criteria, given the high communalities observed (MacCallum et al., 1999). Sample 2 approaches the N ≥ 300 guideline for confirmatory models of this complexity (Wolf et al., 2013). Because both halves derive from a single convenience pool, the split provides internal cross-validation rather than external replication.

### 2.2. Instrument Development and Content Validity

Construct definitions were derived from the intention–behavior gap literature and cognitive dissonance theory (Festinger, 1957; Harmon-Jones & Mills, 2019; Rhodes & de Bruijn, 2013; Sheeran & Webb, 2016). Exercise behavioral inconsistency was defined as a self-perceived, recurring discrepancy between exercise intentions and enacted exercise behavior, together with its affective and justificatory concomitants, and was modeled reflectively across four facets: intention level (IL), intention–action gap (IAG), emotional dissonance (ED), and internal rationalization (IR). The construct was conceptualized as multidimensional rather than as a single homogeneous trait; the four facets index partially separable processes—the formation of intentions, the perceived failure to enact them, the affective response to that failure, and its cognitive neutralization—that need not co-vary within individuals and are therefore better represented as a profile than as a summed total (Rhodes, 2017; Rhodes & Yao, 2015). The instrument’s name emphasizes the inconsistency pole, whereas the instrument deliberately also captures the intention strength and affective valuation that render a lapse consequential; this design choice is examined empirically in Section 3.3 and Section 4.

Items were generated deductively from this literature and refined through expert opinion, following established scale-development guidance (American Educational Research Association et al., 2014; Boateng et al., 2018; Mokkink et al., 2010). An initial pool of 32 items (eight per facet) was reviewed by eight subject matter experts in exercise and sport psychology and scale development, who rated each item for relevance, clarity, and representativeness with respect to its target facet on a four-point relevance scale. Item-level content validity indices (I-CVI), chance-corrected agreement (modified kappa), and the scale-level content validity index (S-CVI/Ave) were computed following Polit et al. (2007); all retained items met the conventional criterion (I-CVI ≥ 0.78), and the S-CVI/Ave met the recommended ≥0.90 standard. Based on this review, the pool was reduced, on content grounds, to the 20 items (5 per facet) administered in the present study. Because the items were generated and curated by the author, target population cognitive pretesting (e.g., think-aloud interviews) was not conducted during development; this is noted as a limitation and a priority for the next validation phase. Items were written and administered in Turkish, each rated on a 5-point Likert scale (1 = strongly disagree, 5 = strongly agree). One item per facet was reverse-worded (IL5, IAG3, ED4, IR3) to attenuate acquiescent responding; these items were reverse-scored before analysis. To address the objectivity of this process, item generation and content validity quantification were kept separate across sources; the eight subject matter experts rated the items independently of the investigator who generated the item pool, and the I-CVI, modified kappa, and S-CVI/Ave were computed directly from those fixed expert ratings, with no additional relevance judgments entering the quantification. Because a single investigator nonetheless coordinated the content validation process, the independent verification of this step is noted as a desirable safeguard (Section Limitations and Future Directions).

### 2.3. Exercise Behavior Indicators

Three single-item indicators of exercise engagement served as criterion variables: weekly exercise frequency (1 = 1–2 days, 2 = 3–4 days, 3 = ≥5 days), average session duration (six ordered categories from 1–15 min to ≥91 min), and exercise experience (six ordered categories from <6 months to >10 years). Given their ordinal metric and their shared self-report method with the ExBIS, associations with ExBIS scores were estimated with Spearman rank correlations and interpreted as mono-method evidence.

### 2.4. Statistical Analyses

Item screening, reliability, and exploratory factor analysis (EFA) were conducted in IBM SPSS Statistics 28 (IBM Corp., Armonk, NY, USA; with O’Connor’s (2000) syntax for parallel analysis and the MAP test); confirmatory factor analysis (CFA), dimensionality modeling, model-based reliability (McDonald’s ω; Dunn et al., 2014; Hayes & Coutts, 2020), average variance extracted, the heterotrait–monotrait ratio (HTMT; Henseler et al., 2015), and measurement invariance were computed in R 4.3.2 (R Foundation for Statistical Computing, Vienna, Austria) using the lavaan (0.6–17) and semTools (0.5–6) packages. There were no missing item-level data. Careless responding was screened using the longstring index (flagging respondents who gave identical answers to all 15 retained items) and Mahalanobis distance (flagging multivariate outliers at *p* < 0.001) (Meade & Craig, 2012); the primary analyses were run on the full sample and re-run with maximal-longstring responders excluded as a sensitivity check. To guard against implementation-specific error, the four-factor maximum-likelihood solution was estimated independently in both environments, which returned identical fit to two decimal places (validation subsample χ^2^(84) = 187.90, CFI = 0.954, TLI = 0.943, RMSEA = 0.065, SRMR = 0.057), confirming that the reported indices are not artifacts of a single program. Because the items are five-category ordinal indicators, the confirmatory model, its subscale reliabilities, and the full configural–metric–scalar invariance sequence were also estimated in this revision with the robust categorical estimator (diagonally weighted least squares with a mean- and variance-adjusted test statistic, WLSMV) on the polychoric correlation matrix, rather than deferring that estimator to future work (Section 3.3 and Section 3.5).

Items were screened on distributional grounds (|skewness| and |kurtosis| < 2) and via corrected item-total correlations (≥0.30). EFA used principal axis factoring with promax rotation (κ = 4) as common-factor extraction and oblique rotation are appropriate for correlated psychological constructs (Costello & Osborne, 2005). Factor retention was decided by the parallel analysis of the reduced correlation matrix, using the common factor variant with 1000 random datasets and a 95th-percentile criterion, supplemented by the minimum average partial (MAP) test (Garrido et al., 2013; Horn, 1965; O’Connor, 2000; Velicer, 1976). The eigenvalue-greater-than-one rule was not used as a retention criterion. Items were retained when the primary pattern loading was ≥0.40 with no secondary loading ≥ 0.32.

Measurement invariance across gender was tested in the configural–metric–scalar sequence, adjudicated by ΔCFI ≤ 0.010 and ΔRMSEA ≤ 0.015 rather than Δχ^2^ alone (Chen, 2007; Cheung & Rensvold, 2002). Because the exercise frequency group is the grouping variable in the known-groups analysis, the identical configural–metric–scalar sequence and adjudication criteria were applied across the three frequency groups, supplemented by Tucker’s coefficient of factor congruence across groups (Lorenzo-Seva & ten Berge, 2006). Known-groups validity was tested with Welch’s ANOVA and Games–Howell post hoc comparisons given heterogeneous variances; because η^2^ is derived from the homoscedastic sum-of-squares partition, Welch-appropriate ε^2^ and ω^2^ are reported as the primary effect sizes alongside η^2^. Gender comparisons used Welch’s *t*-test with Cohen’s *d*. Two-tailed α = 0.05 was used throughout.

## 3. Results

### 3.1. Item Screening

All 20 items showed acceptable univariate distributions; however, the four reverse-worded items displayed weak corrected item-total correlations within their parent subscales (IL5 = 0.27, IAG3 = 0.46, ED4 = 0.32, IR3 = 0.17, after reverse-scoring) and were removed, a pattern consistent with the method variance that reverse-worded items commonly introduce (van Sonderen et al., 2013; Weijters & Baumgartner, 2012). One further item (IR4) was removed during EFA for substantial cross-loading. All analyses below concern the final 15-item version. Item-level descriptive statistics and item-total statistics are presented in Table 2; |skewness| ≤ 1.52 and |excess kurtosis| ≤ 1.80 supported the use of maximum likelihood estimation. Careless-responding screening identified 54 respondents (9.1%) with a maximal long string (identical answers to all 15 items, predominantly the scale midpoint) and 30 respondents (5.1%) exceeding the Mahalanobis criterion (*p* < 0.001). Because the four-factor solution, its fit, and the subscale reliabilities were essentially unchanged when the maximal-longstring cases were excluded (validation-subsample CFA: CFI = 0.953, RMSEA = 0.062; α = 0.74–0.89), the primary analyses retain the full sample, and this exclusion is reported as a sensitivity check. The decision to remove rather than retain the four reverse-worded items was weighed against a method-factor alternative, in which each reverse-worded item would load on its intended facet plus a shared wording/method factor. Two features of the data argue against that account. After reverse-scoring, the items correlated only weakly with their parent facets (corrected item-total *r* = 0.17–0.46), which is the signature of unreliable or conceptually divergent content rather than of otherwise strong indicators carrying additional method variance; each facet formed a clean, well-defined factor once these items were removed. Specifying a wording factor over items this weakly related to their target construct, in a single development subsample, would risk capitalizing on chance. Removal was therefore retained as the more defensible and parsimonious treatment, consistent with evidence that reverse-worded items frequently degrade rather than protect measurement (van Sonderen et al., 2013; Weijters & Baumgartner, 2012).

### 3.2. Exploratory Factor Analysis (Sample 1)

Sampling adequacy was good (KMO = 0.865) and the correlation matrix was factorable, Bartlett’s χ^2^(105) = 2749.83, *p* < 0.001; determinant = 7.7 × 10^−5^. Common-factor parallel analysis retained four factors (fourth real reduced-matrix eigenvalue 0.556 vs. 95th-percentile random 0.282), and the MAP statistic reached its minimum at four components (0.0315), converging on a four-factor solution; the principal-component variant of parallel analysis suggested three factors, illustrating the known sensitivity of retention decisions to the eigenvalue basis and the reliance on the common-factor variant appropriate to principal axis factoring (O’Connor, 2000). The promax-rotated four-factor principal-axis solution accounted for 67.1% of the total variance and displayed a clean, simple structure—primary pattern loadings ranged from 0.56 to 0.96, all secondary loadings were ≤0.24, and communalities ranged from 0.50 to 0.83 (Table 3). Inter-factor correlations were moderate within clusters and negligible between them (|φ| ≤ 0.65), supporting oblique rotation while indicating distinguishable facets; the corresponding latent correlations in the independent validation subsample were smaller (φ = 0.53 for IL with ED and 0.67 for IAG with IR; Section 3.3), and discriminant validity is adjudicated there rather than on the exploratory inter-factor matrix. The factors corresponded exactly to the hypothesized IL, IAG, ED, and IR facets, supporting H1 in the development subsample.

### 3.3. Confirmatory Factor Analysis and Dimensionality (Sample 2)

The correlated four-factor model fit the validation subsample acceptably to well by conventional fit-index cutoffs (Hu & Bentler, 1999; Kline, 2023; Marsh et al., 2004) (χ^2^(84) = 187.90, *p* < 0.001, χ^2^/*df* = 2.24, CFI = 0.954, TLI = 0.943, RMSEA = 0.065, 90% CI [0.052, 0.077], SRMR = 0.057), and decisively outperformed the one-factor model (χ^2^(90) = 1429.77, CFI = 0.408, RMSEA = 0.224 [0.214, 0.235]) and a two-factor model grouping approach (IL + ED) and avoidance (IAG + IR) facets (χ^2^(89) = 588.46, CFI = 0.779, RMSEA = 0.138 [0.127, 0.148]) (Table 4). To test whether the four facets were empirically separable rather than statistical artifacts of correlated factors, two nested three-factor models that collapsed the most strongly correlated pairs of facets were estimated. Merging IAG and IR, the pair with the highest latent correlation (φ = 0.67) significantly degraded fit, Δχ^2^(3) = 74.5, *p* < 0.001, ΔCFI = 0.032, as did merging IL and ED, Δχ^2^(3) = 327.3, *p* < 0.001, ΔCFI = 0.143 (Table 4). Because each merge exceeded the ΔCFI ≤ 0.010 threshold, the four-factor representation was retained over all more parsimonious alternatives.

Crucially, the four facets are not organized by a single higher-order dimension; a second-order model with one general factor over the four first-order factors fit poorly and substantially worse than the correlated model, χ^2^(86) = 286.90, CFI = 0.911, TLI = 0.892, RMSEA = 0.089, SRMR = 0.132, and its second-order loadings were sign-inconsistent (IL = 0.87, ED = 0.60, but IAG = −0.29, IR = −0.13), reflecting that no single factor can reproduce the pattern of strong within-cluster and near-zero between-cluster correlations. A confirmatory bifactor model (Reise, 2012) likewise failed to support an interpretable general dimension; because bifactor solutions are prone to local optima, the model was re-estimated from 60 random starting-value sets, and the best-fitting solution is reported, χ^2^(75) = 184.29, CFI = 0.952, TLI = 0.932, RMSEA = 0.070, SRMR = 0.123. Even at this optimum, explained common variance was only ECV = 0.37 and hierarchical omega ω_H_ = 0.30, both of which are far below the values (ECV ≳ 0.70, ω_H_ ≳ 0.70) that would justify scoring or interpreting a total (Rodriguez et al., 2016), and the general-factor loadings reversed in sign between the IL/ED and IAG/IR clusters (positive for IL and ED, negative for IAG and IR). By contrast, the specific-factor reliabilities were strong for the two commitment facets (ω_HS_ = 0.81 for IL and 0.84 for ED) but low for IAG and IR (ω_HS_ = 0.08 and 0.41), whose reliable variance the bipolar general contrast largely absorbed (within-subscale group-factor ECV = 0.15–0.98); because that general factor reverses in sign across the IL/ED and IAG/IR clusters, it functions as a statistical contrast rather than a substantive common dimension, confirming that the systematic item variance is organized by the facets rather than by a general dimension. The substantial reliability of these two facets is instead evidenced by the correlated four-factor model (ω = 0.90 and 0.78 for IAG and IR) and by their strong first-order loadings, not by the bifactor decomposition. Together, these results establish that ExBIS scores must be interpreted as a four-facet profile rather than summed into a single index; substantively, the facets form two weakly related superordinate clusters—an engagement/commitment cluster (IL with ED) and an inconsistency/neutralization cluster (IAG with IR).

All standardized loadings were significant and substantial (0.66–0.86). Under ML, AVE met the 0.50 benchmark for IL (0.643), IAG (0.670), and ED (0.591), and fell marginally short for IR (0.496), while ML-based composite reliability was adequate to good throughout (IL = 0.88, IAG = 0.89, ED = 0.85, IR = 0.75); because IR’s composite reliability exceeded 0.70 and all its loadings exceeded 0.60, convergent validity can still be considered adequate by the joint criterion (Fornell & Larcker, 1981). Discriminant validity was supported on both criteria—every HTMT value was below 0.85 with its 95% bootstrap interval entirely below the threshold (maximum HTMT = 0.69, 95% CI [0.58, 0.79], for IAG-IR), and the square root of each AVE exceeded that factor’s correlations with all other factors. Latent correlations indicated two coherent clusters, IL with ED (φ = 0.53) and IAG with IR (φ = 0.67), with weak relations between clusters (|φ| ≤ 0.25), replicating H1 in an independent subsample. In direct response to the ordinal estimator concern, the model was re-estimated under the robust categorical WLSMV estimator (polychoric correlations; mean- and variance-adjusted test statistic) in this revision, rather than deferring it. The WLSMV solution reproduced the four-factor structure and preserved the standardized loading pattern (λ = 0.65–0.90); the average variance extracted met the 0.50 criterion for every factor, including IR (IL = 0.76, IAG = 0.75, ED = 0.67, IR = 0.56), and composite reliability under this estimator remained high throughout (IL = 0.90, IAG = 0.90, ED = 0.86, IR = 0.77). Incremental fit under the categorical solution was excellent (CFI = 0.977, TLI = 0.972), and the two-cluster pattern of latent correlations was retained (IL with ED, IAG with IR, near-zero between clusters). These categorical results confirm that IR’s marginally low ML-based AVE is an attenuation artifact of treating five-category ordinal items as continuous (Flora & Curran, 2004) rather than a substantive deficiency, and that the four-factor measurement model is robust to the estimator adopted for the ordinal metric.

### 3.4. Reliability

The three-item IR subscale should be read as provisional throughout this section and the paper; it is the shortest subscale and the least internally consistent (ω = 0.78), and it was also the only facet whose ML-based AVE fell below 0.50 (Section 3.3), making it the instrument’s weakest link for future use (see also Section Limitations and Future Directions).

Internal consistency, estimated on the full sample, was good for IL (ω = 0.88, 95% CI [0.86, 0.90]; α = 0.88), IAG (ω = 0.90, 95% CI [0.89, 0.92]; α = 0.90), and ED (ω = 0.86, 95% CI [0.83, 0.88]; α = 0.85), and acceptable for the three-item IR subscale (ω = 0.78, 95% CI [0.73, 0.81]; α = 0.77), supporting H2. Omega and alpha are reported on the same (full) sample to avoid mixing estimation bases. IR’s omega lower bound remains above 0.70, consistent with the provisional status flagged above.

### 3.5. Measurement Invariance

Across exercise-frequency groups, the grouping variable used for the known-groups analysis, the full configural–metric–scalar sequence, was estimated. The configural model fit well, χ^2^(252) = 419.18, CFI = 0.964, RMSEA = 0.058, SRMR = 0.061, and within-group fit was acceptable to good in every group (CFI = 0.952–0.972, RMSEA = 0.049–0.071). Constraining loadings (metric) produced no deterioration, Δχ^2^(22) = 21.34, *p* = 0.500, ΔCFI = 0.000, ΔRMSEA = −0.003; constraining intercepts (scalar) yielded a statistically significant but trivial change by the oversensitive Δχ^2^ test, Δχ^2^(22) = 45.66, *p* = 0.002, which remained comfortably within the adopted equivalence criteria, ΔCFI = −0.005 (≤0.010) and ΔRMSEA = +0.002 (≤0.015) (Table 5). Full scalar invariance across exercise-frequency groups is therefore supported on the same fit-difference criteria used for gender, and the near-identical standardized loading patterns (Tucker’s congruence φ ≥ 0.995 for all pairwise comparisons) reinforce this conclusion. Consequently, the known-groups observed-mean comparisons reported below are licensed by demonstrated scalar invariance rather than by configural equivalence alone, and the frequency group differences are not attributable to differential item functioning. No latent or observed gender differences emerged; all subscale comparisons were nonsignificant, including ED, which approached but did not reach the criterion, *t*(503.0) = 1.96, *p* = 0.051, *d* = 0.17. The full configural–metric–scalar sequence was additionally re-estimated under the robust categorical WLSMV estimator (polychoric correlations; mean- and variance-adjusted test statistic), and this solution reproduced the maximum-likelihood result—full scalar invariance was retained across both gender and exercise-frequency groups, with every multigroup change in fit within the adopted equivalence criteria (ΔCFI ≤ 0.010, ΔRMSEA ≤ 0.015). Specifically, the categorical sequence constrained loadings at the metric step and loadings together with item thresholds at the scalar step, thresholds rather than intercepts being the relevant location parameters for ordered-categorical indicators (Wu & Estabrook, 2016); across gender the two steps yielded ΔCFI = +0.001 and ΔRMSEA = −0.004 (metric) and ΔCFI = −0.001 and ΔRMSEA = −0.007 (scalar), and across exercise-frequency groups ΔCFI = +0.001 and ΔRMSEA = −0.006 (metric) and ΔCFI = +0.002 and ΔRMSEA = −0.011 (scalar), with good configural fit for both groupings (scaled CFI = 0.973).

### 3.6. Known-Groups and Criterion Validity

Welch’s ANOVA was significant for every facet—IL, *F*(2, 323.0) = 19.79, *p* < 0.001, ω^2^ = 0.064; IAG, *F*(2, 314.3) = 25.48, *p* < 0.001, ω^2^ = 0.081; ED, *F*(2, 324.7) = 21.77, *p* < 0.001, ω^2^ = 0.066; and IR, *F*(2, 312.8) = 10.57, *p* < 0.001, ω^2^ = 0.031 (Table 6). Games–Howell contrasts showed that IL and ED increased monotonically with exercise frequency (1–2 days < 3–4 days < ≥5 days, all *p* ≤ 0.004), while IAG decreased monotonically (1–2 days > 3–4 days > ≥5 days, all *p* ≤ 0.010). IR was elevated only in the least active group (1–2 days > 3–4 days = ≥5 days; the contrast between the two more active groups was non-significant, *p* = 0.501), so its group effect is driven by the low-frequency group rather than by a graded trend. Effect sizes were small to medium throughout. All four subscales also correlated with weekly frequency, average session duration, and exercise experience in the predicted directions (ρ = 0.15 to 0.30 in absolute value, all *p* < 0.001), with IL and ED correlating positively and IAG and IR negatively (Table 6).

The strongest criterion association involved IAG (ρ = −0.30 with frequency). Because the IAG items refer explicitly to planning to exercise and failing to follow through, this association partly reflects definitional proximity to the frequency criterion rather than purely external validity, and is interpreted with that caveat. To gauge the extent of this content overlap, the intention-level association with frequency was recomputed after removing its most behaviorally worded item (IL2, “I intend to exercise regularly several days a week”); the correlation was essentially unchanged (ρ = 0.27 with all four items vs. 0.26 without IL2), and the affective (ED) and justificatory (IR) facets, whose content is not behavioral, retained their associations with frequency (ρ = 0.26 and –0.19). The known-groups pattern is therefore not reducible to definitional overlap except in the specific and acknowledged case of the IAG subscale, whose behavioral content makes a degree of criterion proximity unavoidable. Together these results support H4 and provide partial support for H5, with the qualifications that the IAG frequency association is partly definitional, and that the IR’s group effect was driven by the low-frequency group.

## 4. Discussion

This study developed ExBIS and assembled initial evidence of its internal structure, reliability, measurement invariance, and relations to exercise behavior. Across independent development and validation subsamples, the data supported a correlated four-factor structure, IL, IAG, ED, and IR, that was reproduced under exploratory and confirmatory analysis, survived comparison against more parsimonious alternatives, and was robust to a categorical treatment of the items and to the exclusion of careless responders. The four facets are best understood as a profile rather than as components of a single trait, a point developed below because it has direct implications for how the instrument should be scored and interpreted.

The central structural finding is that the four facets are distinguishable but are not organized by a single overarching dimension. Sequentially merging the most strongly correlated factor pairs significantly degraded fit, confirming that the facets are empirically separable rather than redundant. At the same time, neither a second-order model nor a bifactor model recovered an interpretable general factor; the second-order solution fit poorly and produced sign-inconsistent higher-order loadings, and the bifactor general factor explained little common variance (ECV = 0.37) and carried negligible score-relevant reliability (ω_H_ = 0.30), with loadings that reversed in sign between the IL/ED and IAG/IR clusters. The practical implication is unambiguous and important to state plainly: ExBIS items should not be summed into a total score. The instrument yields a four-facet profile, and the facets cohere into two weakly related superordinate clusters—an engagement/commitment cluster (IL and ED) and an inconsistency/neutralization cluster (IAG and IR). This two-cluster architecture is theoretically sensible; individuals who value exercise and feel discomfort when they miss it (high IL, high ED) are not necessarily the same individuals who report a large enacted gap and justify it (high IAG, high IR), and the near-zero between-cluster correlations indicate that commitment and inconsistency are separable rather than opposite ends of one continuum. The instrument’s name foregrounds the inconsistency pole; the evidence here clarifies that the ExBIS in fact characterizes the broader cognitive–affective configuration in which inconsistency is embedded, and the name should be read with that scope in mind.

The facets are joined by functional dependency rather than by covariance. A discrepancy is undefined in the absence of an intention, so IAG presupposes IL rather than merely correlating with it; dissonance arises only where a valued cognition has been violated, so ED is uninterpretable without commitment; and IR is, by definition, the neutralization of that dissonance. On this analysis, the near-independence of the two clusters is a requirement of the design rather than a weakness in it. Were IL and the IAG strongly related, a respondent’s profile would carry no information beyond a single dimension, and the instrument’s stated purpose, distinguishing individuals who share the same enacted gap but differ in how they experience and neutralize it, would be unachievable. The configuration is therefore the unit of measurement, and the instrument’s name marks the pole of applied interest rather than the whole of what is measured. This position carries an evidential burden the present cross-sectional design cannot discharge; it requires demonstrating that facet configurations predict outcomes the facets do not predict individually, which is a task for the prospective work outlined in Section Limitations and Future Directions.

The ExBIS is scored as four separate subscale means (or sums), IL, IAG, ED, and IR, each interpreted on its own metric; no total score is computed. Critically, the two superordinate clusters identified here (IL with ED; IAG with IR) are interpretive groupings that describe how the facets covary, not scoring units; because the within-cluster correlations are only moderate (φ = 0.53 and 0.67) and the four facets remain empirically distinct (every merged model degraded fit and every HTMT interval fell below 0.85), the subscales should not be collapsed into two cluster composites any more than into a single total. A respondent’s ExBIS result is therefore a four-value profile, and any lower-dimensional summary should describe the two clusters qualitatively rather than average across them.

Reliability and convergent/discriminant evidence supported the four-facet model. Omega and alpha, reported on a common sample to avoid mixing estimation bases, were good for IL, IAG, and ED and acceptable for the briefer IR subscale. Discriminant validity held on both the Fornell–Larcker criterion and the heterotrait–monotrait ratio, with every HTMT estimate and its bootstrap interval below the 0.85 threshold. IR was the weakest facet; its average variance extracted fell marginally short of 0.50 under maximum likelihood, though it met the criterion when the ordinal item metric was modeled via polychoric correlations, and it is the shortest subscale. IR is therefore treated as provisional, and the addition of one to two items is flagged as a priority for the next revision.

The pattern of relations to exercise behavior was consistent with theoretical expectations. All four facets distinguished exercise frequency groups in the predicted directions, and subscale scores correlated with frequency, session duration, and exercise experience. These known-groups comparisons are interpretable because the four-factor model showed full scalar invariance across the frequency groups (ΔCFI ≤ 0.005, ΔRMSEA ≤ 0.003), so the observed mean differences reflect genuine latent differences rather than non-equivalent measurement or differential item functioning. Two interpretive cautions temper these associations. First, the effect sizes were small to medium (ω^2^ = 0.031–0.081), as expected for self-report trait-behavior relations, and all criterion variables share the self-report method of the predictor. Second, the IAG subscale refers explicitly to planning to exercise and failing to follow through, so its association with the frequency criterion is partly definitional; a sensitivity check confirmed that the remaining facets’ associations were not driven by such overlap (IL association was unchanged after removing its most behavioral item, and the affective and justificatory facets, whose content is not behavioral, retained their associations). For these reasons, H5 is regarded as partially rather than fully supported.

ED, as operationalized here, also overlaps conceptually with anticipated guilt and affective commitment; several ED items reference guilt and self-directed frustration explicitly. Empirically distinguishing ED from these neighboring constructs is therefore a central task for the convergent study described below. ED is predicted a priori to correlate moderately, but not redundantly, with anticipated exercise guilt (an expected r in the 0.40–0.60 range) while remaining empirically separable from it (HTMT < 0.85); confirming this specific discriminant prediction is necessary before ED can be claimed as more than a relabeling of exercise guilt.

A profile marked by high intention, a large enacted gap, and high rationalization is a plausible signature of the “unsuccessful intender” for whom action planning and self-regulatory support may be more appropriate than motivational enhancement (Sniehotta et al., 2005). Such a profile could accordingly help identify individuals at elevated risk of failing to maintain exercise. On the same logic, the facet pattern could inform which behavior-changing strategy to prioritize—self-regulatory and action planning support for high-gap, high-rationalization profiles versus motivational or value-clarification work for low-intention profiles—thereby assisting clinicians and exercise professionals in matching individuals to interventions, and repeated administration could, in principle, help monitor cognitive–affective change across a behavior change or rehabilitation program. Each of these is a hypothesis the instrument enables rather than a demonstrated property; whether such profiles actually forecast subsequent disengagement, and therefore whether the ExBIS has utility as a screening or triage tool, is an empirical question that presupposes the prospective, longitudinal predictive evidence the present cross-sectional design does not provide, and none of these applications should guide clinical or applied decision-making until that evidence is established.

### Limitations and Future Directions

Several limitations qualify these conclusions and define the next phase of validation. First, and most consequential, the validity evidence assembled here concerns internal structure, reliability, and known-groups and criterion relations with exercise indicators; the study did not include established self-report instruments, so convergent and discriminant validity against existing measures remain to be established. This is the single most important priority for subsequent work. A focused convergent battery should situate each facet against its nearest established neighbors—IL against theory-of-planned-behavior intention measures, IAG against action control and behavioral regulation indices, ED against anticipated guilt and affective-commitment scales, and IR against measures of exercise excuses and self-handicapping, with a priori predictions about which correlations should be strong and which weak. Until that evidence exists, the present results should be read as an initial structural and reliability validation rather than a comprehensive one, and applied use is premature.

Second, the design was cross-sectional and single-source. Test–retest reliability and the temporal stability of the profile were not assessed. All constructs and criteria shared the self-report method, so common method variance cannot be excluded (Podsakoff et al., 2003). Although the catastrophic fit of the one-factor model argues against a single pervasive method factor, that test addresses only a dominant general factor and does not preclude method variance inflating specific scale-criterion associations, so a confirmatory check using an unmeasured latent method factor or marker variable is recommended. Prospective designs that relate baseline ExBIS profiles to device-measured or longitudinally tracked exercise behavior are needed both to establish predictive validity and to test the mechanistic hypotheses described above. Third, the four-factor structure was estimated under maximum likelihood and corroborated under the robust categorical WLSMV estimator (polychoric correlations; mean- and variance-adjusted test statistic), under which incremental fit was excellent (CFI = 0.977), every factor including IR met the 0.50 average-variance-extracted criterion, and full scalar invariance was reproduced across both gender and exercise-frequency groups. These categorical re-estimations were carried out in the present revision rather than deferred; nonetheless, a WLSMV implementation in a dedicated structural equation-modeling program remains the reference estimator for five-category ordinal items, and independent replication of the full model and invariance sequence in such a program and in larger, more diverse samples remains desirable.

Fourth, the sample was a convenience sample of predominantly young Turkish adults recruited online, and the development and validation subsamples were random halves of a single pool, providing internal cross-validation rather than external replication. Social-media recruitment yields a self-selected sample over-representing younger, more digitally engaged, and university-educated respondents. It should not be treated as representative of the broader adult population, and generalization to older adults, clinical populations, and other cultural and linguistic contexts requires independent samples. The forced-response format, while eliminating item-level missingness, removed the option to decline individual items and contributed to a non-trivial rate of maximal-longstring responding (9.1%); although the factor structure and reliabilities were robust to excluding these cases, future administrations should embed validated attention-check and instructed-response items in the primary design, rather than relying on post-hoc screening, and should consider supervised or panel-based recruitment. Because a single investigator generated the items, coordinated the content-validity assessment, collected the data, and analyzed them without preregistration, the analytic choices, though specified a priori and reproducible from the syntax available on request, carry investigator degrees of freedom that preregistration and independent replication would constrain.

Fifth, the scale was developed and validated only in Turkish; the English wording in Table 7 is a working translation that has not undergone independent forward–back translation or cross-cultural validation, and cross-lingual use should follow established translation and invariance procedures. Finally, the IR item pool is thin, and its average variance extracted is borderline below the maximum-likelihood threshold (adequate under the categorical solution); expanding and revalidating this facet, accompanied by the target population cognitive pretesting not undertaken at the development stage, is a concrete near-term step.

## 5. Conclusions

ExBIS provides initial evidence for a psychometrically defensible, multidimensional profile of the cognitive–affective architecture surrounding the exercise intention–behavior gap. Its four facets, IL, IAG, ED, and IR, are distinguishable, reliable, and invariant across gender, and they relate to exercise behavior in theoretically coherent ways. Because the facets are not organized by a single general factor, the instrument should be scored and interpreted as a profile across two weakly related dimensions rather than as a single total. The ExBIS thereby offers researchers a measurement tool for constructs that post-intentional theories treat as central but that have lacked dedicated assessment. Convergent validation against established instruments, test–retest assessment, prospective tests of predictive and mechanistic hypotheses, and cross-cultural extension are the necessary next steps before the instrument is used in applied settings. Because they rest on a single cross-sectional convenience sample of predominantly young Turkish adults assessed in a single language, the present findings constitute only initial validation evidence; external validation in independent, more diverse samples, longitudinal investigation of predictive validity, and cross-cultural replication are prerequisites for any widespread or applied implementation of the ExBIS.

## Figures and Tables

**Table 1:** Sample characteristics.

Characteristic	Total (*N* = 594)	Sample 1 (*n* = 297)	Sample 2 (*n* = 297)
Age, *M* (*SD*)	21.02 (4.87)	20.81 (4.41)	21.23 (5.29)
Gender, *n* (%) male	344 (57.9)	163 (54.9)	181 (60.9)
Education, *n* (%)			
High school	178 (30.0)	—	—
Associate degree	54 (9.1)	—	—
Bachelor	342 (57.6)	—	—
Postgraduate	20 (3.4)	—	—
Weekly exercise frequency, *n* (%)		—	—
1–2 days	175 (29.5)	—	—
3–4 days	283 (47.6)	—	—
≥5 days	136 (22.9)	—	—

*Note.* Samples 1 and 2 are random halves of the full sample (seed = 31,216) used for exploratory and confirmatory analyses, respectively, and did not differ in age or gender composition. Em dashes (—) indicate that the variable was not tabulated separately by subsample; they do not denote zero counts.

**Table 2:** Item descriptive and item-total statistics (*N* = 594).

Item	*M*	*SD*	Skew.	Kurt.	*r*(it)	α If Del.
IL1	4.25	0.98	−1.23	0.88	0.80	0.82
IL2	4.27	1.02	−1.38	1.29	0.76	0.83
IL3	4.40	0.94	−1.52	1.80	0.75	0.84
IL4	4.12	1.11	−1.09	0.37	0.65	0.88
IAG1	2.49	1.32	0.45	−0.83	0.75	0.89
IAG2	2.45	1.40	0.51	−0.99	0.81	0.87
IAG4	2.68	1.34	0.24	−1.05	0.77	0.88
IAG5	2.40	1.31	0.55	−0.78	0.80	0.87
ED1	3.69	1.27	−0.64	−0.57	0.64	0.84
ED2	3.71	1.21	−0.57	−0.58	0.77	0.78
ED3	3.72	1.19	−0.52	−0.64	0.75	0.79
ED5	3.67	1.25	−0.55	−0.73	0.63	0.84
IR1	3.20	1.16	−0.07	−0.59	0.61	0.69
IR2	2.93	1.24	0.15	−0.83	0.66	0.62
IR5	2.73	1.27	0.24	−0.83	0.55	0.76

*Note. r*(it) = corrected item-total correlation within the parent subscale. Skew. = skewness; Kurt. = excess kurtosis. A longstring screen flagged 54 respondents (9.1%) who answered all 15 items identically (predominantly the midpoint) and 30 (5.1%) exceeding the Mahalanobis criterion (*p* < 0.001); excluding maximal-longstring cases left the factor structure and reliabilities essentially unchanged.

**Table 3:** Exploratory factor analysis: promax-rotated pattern matrix (Sample 1, *n* = 297).

Item	F1 (IL)	F2 (IAG)	F3 (ED)	F4 (IR)	*h* ^2^
IL1	0.93	0.12	–0.00	–0.04	0.82
IL2	0.78	–0.02	0.01	0.05	0.64
IL3	0.87	0.04	–0.06	0.05	0.69
IL4	0.69	–0.09	0.04	–0.04	0.55
IAG1	0.04	0.79	–0.00	0.04	0.65
IAG2	–0.02	0.92	0.02	–0.06	0.79
IAG4	0.08	0.87	–0.03	–0.00	0.73
IAG5	–0.05	0.85	0.03	0.02	0.77
ED1	0.21	–0.12	0.56	0.11	0.54
ED2	–0.07	0.02	0.96	–0.00	0.83
ED3	–0.11	0.01	0.92	0.00	0.72
ED5	0.18	0.09	0.58	–0.11	0.50
IR1	0.09	–0.03	0.00	0.80	0.63
IR2	–0.02	–0.01	–0.03	0.82	0.66
IR5	–0.08	0.24	0.03	0.56	0.54
Reduced-matrix eigenvalue	4.54	3.67	0.98	0.56	
PA criterion (95th pctl)	0.55	0.43	0.35	0.28	

*Note.* Primary loadings in bold in the published version. PA = parallel analysis (common-factor variant, 1000 datasets). Extraction was performed by principal axis factoring with Kaiser-normalized promax rotation (κ = 4); *h*^2^ are extraction communalities. The four retained factors explained 67.1% of the total variance.

**Table 4:** Confirmatory factor analysis: fit of the hypothesized model and alternatives (Sample 2, *n* = 297).

Model	χ^2^ (*df*)	CFI	TLI	RMSEA [90% CI]	SRMR	Δχ^2^ (Δ*df*)	ΔCFI
Four-factor (hypothesized)	187.90 (84)	0.954	0.943	0.065 [0.052, 0.077]	0.057	—	—
Three-factor (IAG + IR merged)	262.40 (87)	0.922	0.906	0.083 [0.071, 0.094]	0.065	74.5 (3) ***	0.032
Three-factor (IL + ED merged)	515.16 (87)	0.811	0.772	0.129 [0.119, 0.140]	0.094	327.3 (3) ***	0.143
Two-factor (IL + ED/IAG + IR)	588.46 (89)	0.779	0.740	0.138 [0.127, 0.148]	0.099	399.9 (5) ***	0.175
One-factor	1429.77 (90)	0.408	0.310	0.224 [0.214, 0.235]	0.212	1241.2 (6) ***	0.546
Second-order (one general factor)	286.90 (86)	0.911	0.892	0.089 [0.078, 0.101]	0.132	99.00 (2) ***	0.043
Bifactor (general + 4 group)	184.29 (75)	0.952	0.932	0.070 [0.057, 0.083]	0.123	—	—

*Note.* Δχ^2^ and ΔCFI for the merged and reduced models are relative to the four-factor model; the second-order comparison is also relative to the four-factor model (Δ*df* = 2). The bifactor model was estimated from 60 random starting-value sets, and the best-fitting solution is reported. Explained common variance ECV = 0.37 and hierarchical omega ω_H_ = 0.30 indicate that no interpretable general factor underlies the items, so a total score is not justified; by contrast, the specific-factor reliabilities were strong for the commitment facets (ω_HS_ = 0.81 and 0.84 for IL and ED) but low for IAG and IR (0.08 and 0.41), whose reliable variance the bipolar general contrast largely absorbs (within-subscale group-factor ECV = 0.15–0.98). A categorical (polychoric) re-estimation preserved the loading pattern with AVE ≥ 0.50 for every factor. Under this ordinal parameterization, the robust WLSMV solution reported in Section 3.3 and Section 3.5 yielded CFI = 0.977, TLI = 0.972, while an unweighted-least-squares (cat-ULS) solution on the same matrix yielded CFI = 0.984, TLI = 0.980. Em dashes (—) indicate that a comparison is not applicable. *** *p* < 0.001.

**Table 5:** Measurement invariance across gender and across exercise frequency groups (full sample).

Model	χ^2^ (*df*)	CFI	RMSEA	SRMR	Δχ^2^ (Δ*df*), *p*	ΔCFI	ΔRMSEA
**Maximum likelihood**							
Gender-configural	324.80 (168)	0.968	0.056	0.058	—	—	—
Gender-metric	334.60 (179)	0.968	0.054	0.060	9.79 (11), 0.549	0.000	−0.002
Gender-scalar	345.80 (190)	0.968	0.053	0.060	11.20 (11), 0.427	0.000	−0.001
Frequency-configural	419.18 (252)	0.964	0.058	0.061	—	—	—
Frequency-metric	440.52 (274)	0.964	0.055	0.065	21.34 (22), 0.500	0.000	−0.003
Frequency-scalar	486.18 (296)	0.959	0.057	0.065	45.66 (22), 0.002	−0.005	+0.002
**WLSMV (categorical)**							
Gender-configural	542.06 (168)	0.973	0.087	0.065	—	—	—
Gender-metric	546.08 (179)	0.974	0.083	0.067	19.75 (11), 0.049	+0.001	−0.004
Gender-scalar	596.71 (220)	0.973	0.076	0.066	41.90 (41), 0.432	−0.001	−0.007
Frequency-configural	600.22 (252)	0.973	0.084	0.068	—	—	—
Frequency-metric	605.58 (274)	0.974	0.078	0.070	39.34 (22), 0.013	+0.001	−0.006
Frequency-scalar	672.39 (356)	0.976	0.067	0.068	41.10 (82), 1.000	+0.002	−0.011

*Note.* Metric = equal loadings; scalar = equal loadings and intercepts (ML) or equal loadings and thresholds (WLSMV; Wu & Estabrook, 2016). For the WLSMV models, Δχ^2^ is the scaled (robust) difference test, and the configural row is the baseline (—). Invariance was adjudicated on ΔCFI ≤ 0.010 and ΔRMSEA ≤ 0.015 (Chen, 2007; Cheung & Rensvold, 2002) rather than the oversensitive χ^2^ difference, which flags trivial metric step differences under WLSMV. SRMR is the no-mean-structure variant; WLSMV fit indices are the scaled (robust) values. Full scalar invariance held for both groupings under both estimators; within-group configural fit was acceptable to good in every frequency group (CFI = 0.952–0.972), and Tucker’s congruence exceeded 0.995 for all pairwise comparisons. Bold row labels (Maximum likelihood; WLSMV [categorical]) are panel headings identifying the estimator used for the models listed beneath them; em dashes (—) denote comparisons that are not applicable to the baseline configural model.

**Table 6:** Known-groups differences across exercise frequency groups and criterion correlations (full sample).

Subscale	Welch *F* (*df*1, *df*2)	ω^2^ (η^2^)	Pattern	ρ Freq.	ρ Dur.	ρ Exp.
IL	19.79 (2, 323.0) ***	0.064 (0.067)	1 < 2 < 3	0.27 ***	0.29 ***	0.15 ***
IAG	25.48 (2, 314.3) ***	0.081 (0.084)	1 > 2 > 3	−0.30 ***	−0.27 ***	−0.25 ***
ED	21.77 (2, 324.7) ***	0.066 (0.069)	1 < 2 < 3	0.26 ***	0.24 ***	0.16 ***
IR	10.57 (2, 312.8) ***	0.031 (0.035)	1 > 2 = 3	−0.19 ***	−0.16 ***	−0.16 ***

*Note.* Groups: 1 = 1–2 days, 2 = 3–4 days, 3 = ≥5 days. ω^2^ and ε^2^ (Welch-appropriate effect sizes) were identical to three decimals and are summarized by ω^2^; η^2^ is reported in parentheses for comparison. Pattern reflects significant Games–Howell contrasts. ρ = Spearman correlation; Freq. = weekly frequency, Dur. = session duration, Exp. = exercise experience. *** *p* < 0.001.

**Table 7:** Final 15-item ExBIS with administered (Turkish) wording and a working English translation.

Item	English Wording (Non-Validated Working Translation)	Turkish Wording (Administered)
IL1	I want to make exercise a part of my lifestyle.	Egzersiz yapmayı yaşam tarzımın bir parçası haline getirmek istiyorum.
IL2	I intend to exercise regularly several days a week.	Haftada birkaç gün düzenli egzersiz yapma niyetindeyim.
IL3	I believe in the long-term health benefits of exercise.	Egzersiz yapmanın uzun vadeli sağlık yararlarına inanıyorum.
IL4	I feel motivated to exercise.	Egzersiz yapma konusunda motive hissediyorum.
IAG1	I plan to exercise but often do not follow through.	Egzersiz yapmayı planlarım ama çoğu zaman uygulamam.
IAG2	I often say “I’ll start tomorrow” and postpone it.	“Yarın kesin başlıyorum” diyerek ertelerim.
IAG4	Even though my intention is strong, I struggle to act on it.	Niyetim güçlü olsa da harekete geçmekte zorlanırım.
IAG5	I frequently delay my decisions to exercise.	Egzersiz yapma kararlarımı çoğunlukla ertelerim.
ED1	I feel uneasy when I don’t exercise.	Egzersiz yapmadığımda kendimi rahatsız hissederim.
ED2	I feel guilty when I don’t exercise enough.	Yeterince egzersiz yapmadığım zaman vicdan azabı duyarım.
ED3	I get angry with myself when I skip exercise.	Egzersiz yapmayınca kendime kızarım.
ED5	When I neglect exercise, thinking about it bothers me.	Egzersizi ihmal ettiğimde, bunu düşünmek canımı sıkar.
IR1	It’s normal not to exercise when I don’t have time.	Zamanım yoksa egzersiz yapmamam normaldir.
IR2	If I’m tired, skipping exercise is my right.	Yorgunsam egzersizi atlamak benim hakkımdır.
IR5	I use busy days as a reason not to exercise.	Yoğun günlerimi egzersiz yapmamak için gerekçe olarak görürüm.

*Note.* Items were administered in Turkish on a 5-point Likert scale (1 = strongly disagree, 5 = strongly agree). The English wording is a non-validated working translation provided for the reader; it has not undergone independent forward–back translation or cross-cultural validation.

## Data Availability

The data presented in this study are available on request from the corresponding author; the full dataset was provided to the journal during peer review for editorial verification.
